# *Notes from the Field:* E-Cigarette Use Among Middle and High School Students — National Youth Tobacco Survey, United States, 2021

**DOI:** 10.15585/mmwr.mm7039a4

**Published:** 2021-10-01

**Authors:** Eunice Park-Lee, Chunfeng Ren, Michael D. Sawdey, Andrea S. Gentzke, Monica Cornelius, Ahmed Jamal, Karen A. Cullen

**Affiliations:** ^1^Center for Tobacco Products, Food and Drug Administration, Silver Spring, Maryland; ^2^Office on Smoking and Health, National Center for Chronic Disease Prevention and Health Promotion, CDC.

Since 2014, e-cigarettes have been the most commonly used tobacco product among U.S. youths (*1*). In 2020, an estimated 3.6 million (13.1%) U.S. middle and high school students reported using e-cigarettes within the past 30 days (current use); more than 80% of current users reported flavored e-cigarette use (*2*). Whereas the most commonly used device type in 2019 and 2020 was a prefilled pod or cartridge,* disposable e-cigarette use increased significantly during this time among youths who currently used e-cigarettes in middle school (from 3.0% to 15.2%) and high school (from 2.4% to 26.5%) (*3*). CDC and the Food and Drug Administration (FDA) analyzed nationally representative data from the 2021 National Youth Tobacco Survey (NYTS), a school-based, cross-sectional, self-administered survey of U.S. middle school (grades 6–8) and high school (grades 9–12) students conducted during January 18–May 21, 2021 (20,413 students from 279 schools; overall response rate = 44.6%).^†^ Because of the ongoing COVID-19 pandemic, data were collected online to allow participation of eligible students in remote learning settings.^§^ Current e-cigarette use was assessed overall, by frequency of use, device type, flavors, and usual brand. Weighted prevalence estimates and population totals^¶^ were calculated. This study was reviewed and approved by the CDC IRB.**

In 2021, 11.3% of high school students (1.72 million) and 2.8% (320,000) of middle school students reported current e-cigarette use (Table). Among current e-cigarette users, 43.6% of high school students and 17.2% of middle school students reported using e-cigarettes on ≥20 of the past 30 days; daily use was 27.6% among current high school e-cigarette users and 8.3% among current middle school e-cigarette users. Among both middle and high school current e-cigarette users, the most commonly used device type was disposables, followed by prefilled or refillable pods or cartridges and tanks or mod systems. Among high school current e-cigarette users, 26.1% reported that their usual brand was Puff Bar, followed by Vuse (10.8%), SMOK (9.6%), JUUL (5.7%), and Suorin (2.3%). Among middle school current users, 30.3% reported that their usual brand was Puff Bar, and 12.5% reported JUUL. Notably, 15.6% of high school users and 19.3% of middle school users reported not knowing the e-cigarette brand they usually used.

**TABLE:** Prevalence of past 30-day e-cigarette use,* overall and by selected characteristics and school level — National Youth Tobacco Survey, United States, 2021

Characteristic	Overall		High school		Middle school	
	% (95% CI)	Estimated weighted no.^†^	% (95% CI)	Estimated weighted no.^†^	% (95% CI)	Estimated weighted no.^†^
**Among all students**						
Current use of e-cigarettes	7.6 (6.6–8.7)	2,060,000	11.3 (9.7–13.0)	1,720,000	2.8 (2.2–3.4)	320,000
**Among current e-cigarette users**						
**Frequency of e-cigarette use**						
1–19 days per month	60.6 (56.5–64.6)	1,240,000	56.4 (51.8–61.0)	970,000	82.8 (77.4–87.2)	270,000
20–30 days per month	39.4 (35.4–43.5)	810,000	43.6 (39.0–48.2)	750,000	17.2 (12.8–22.6)	50,000
**Daily e-cigarette use^§^**	24.6 (21.8–27.8)	500,000	27.6 (24.3–31.2)	470,000	8.3 (5.6–12.0)	20,000
**Device type used^¶^**						
Disposables	53.7 (48.7–58.6)	1,080,000	55.8 (50.8–60.7)	940,000	43.8 (34.0–54.1)	130,000
Prefilled or refillable pods or cartridges	28.7 (25.1–32.6)	570,000	28.9 (24.9–33.3)	480,000	27.8 (22.0–34.4)	80,000
Tanks or mod systems	9.0 (6.8–11.8)	180,000	7.5 (5.5–10.3)	120,000	15.6 (9.7–24.1)	40,000
Don’t know	8.6 (6.7–11.0)	170,000	7.8 (5.7–10.4)	130,000	12.8 (8.0–19.9)	40,000
Usual brand**						
Puff Bar	26.8 (22.9–31.1)	520,000	26.1 (22.0–30.6)	430,000	30.3 (21.9–40.3)	90,000
Vuse	10.5 (6.9–15.6)	200,000	10.8 (7.1–16.2)	170,000	—^††^	—
SMOK (including NOVO)	8.6 (6.4–11.5)	160,000	9.6 (7.1–13.0)	150,000	—	—
JUUL	6.8 (4.9–9.3)	130,000	5.7 (3.8–8.5)	90,000	12.5 (8.3–18.4)	30,000
Suorin	2.1 (1.2–3.7)	40,000	2.3 (1.3–4.0)	30,000	—	—
No usual brand	2.4 (1.5–3.8)	40,000	2.5 (1.5–4.1)	40,000	—	—
Some other brand not listed	19.8 (15.7–24.6)	390,000	21.0 (16.5–26.3)	340,000	13.8 (8.6–21.3)	40,000
Don’t know	16.1 (13.8–18.8)	310,000	15.6 (13.1–18.4)	250,000	19.3 (14.2–25.8)	60,000
**Flavored e-cigarette use^§§^**						
Yes	84.7 (81.4–87.5)	1,680,000	85.8 (82.3–88.7)	1,420,000	79.2 (69.1–86.6)	250,000
No	8.8 (6.9–11.2)	170,000	8.4 (6.5–10.7)	130,000	11.1 (6.4–18.7)	30,000
Don’t know	6.5 (5.0–8.4)	120,000	5.9 (4.3–8.0)	90,000	9.7 (6.3–14.7)	30,000
**Flavor type used^¶¶^**						
Fruit	71.6 (67.8–75.1)	1,190,000	72.3 (68.1–76.1)	1,010,000	68.1 (58.7–76.1)	160,000
Candy, desserts, or other sweets	34.1 (30.3–38.2)	560,000	33.0 (29.2–37.1)	460,000	38.8 (30.0–48.3)	90,000
Mint	30.2 (26.9–33.7)	500,000	30.5 (27.0–34.2)	420,000	26.7 (19.5–35.4)	60,000
Menthol	28.8 (23.6–34.8)	470,000	29.8 (24.2–36.0)	410,000	23.1 (13.8–36.0)	50,000
Alcoholic drink	6.0 (4.3–8.2)	90,000	5.0 (3.4–7.5)	70,000	10.3 (5.9–17.3)	20,000
Chocolate	2.9 (1.9–4.5)	40,000	2.5 (1.4–4.4)	30,000	—	—
Clove or spice	2.1 (1.3–3.3)	30,000	—	—	—	—
Some other flavor not listed	10.4 (8.2–13.2)	170,000	9.8 (7.4–12.7)	130,000	13.8 (8.5–21.6)	30,000

**Abbreviation:** CI = confidence interval.

* Past 30-day use of e-cigarettes was determined by asking, “During the past 30 days, on how many days did you use e-cigarettes?” Current use was defined as use on ≥1 day during the past 30 days.

^†^ Estimated total number of users was rounded down to the nearest 10,000 persons. Overall population totals might not sum to corresponding estimates by school level because of rounding or inclusion of students who did not self-report their grade level.

^§^ Daily e-cigarette use was defined as reported use on all 30 days during the past 30 days.

^¶^ Device type use among current e-cigarette users was determined by answers to the question, “Which of the following best describes the type of e-cigarette you have used in the past 30 days? If you have used more than one type, please think about the one you use most often.” Response options were the following: “a disposable e-cigarette (for example, Puff Bar, STIG),” “an e-cigarette that uses pre-filled or refillable pods or cartridges (for example, JUUL, SMOK, or Suorin),” “an e-cigarette with a tank that you refill with liquids (including mod systems that can be customized by the user),” and “I don’t know the type.”

** Usual brand was determined by two questions. All current e-cigarette users were first asked, “During the past 30 days, what e-cigarette brands did you use? (Select one or more).” Response options were as follows: “blu,” “Eonsmoke,” “JUUL,” “Leap,” “Logic,” “Mojo,” “NJOY,” “Posh,” “Puff Bar,” “SMOK (including NOVO),” “STIG,” “Suorin,” “Vuse,” “Some other brand(s) not listed here,” and “Not sure/I don’t know the brand.” Those who selected more than one option were then asked, “During the past 30 days, what brand of e-cigarettes did you usually use? (Choose only one answer).” The same response options as the first question were available with the additional response option of “I did not use a usual brand.” If a single brand was selected in the first question, that brand was reported as their usual brand. Otherwise, the option selected in the second question was recorded as the usual brand. Those who selected “Some other brand(s) not listed here” could provide a write-in response; write-in responses corresponding to an original response option were recoded. Data for blu, Eonsmoke, Leap, Logic, Mojo, NJOY, Posh, and STIG are not shown because of statistically unreliable estimates resulting from an unweighted denominator <50 or a relative standard error >30% overall and at both school levels.

^††^ Dashes indicate data were statistically unreliable because of an unweighted denominator <50 or a relative standard error >30%.

^§§^ Flavored e-cigarette use was assessed by the question, “Were any of the e-cigarettes that you used in the past 30 days flavored to taste like menthol, mint, clove or spice, alcohol drinks, candy, fruit, chocolate, or any other flavor?” Responses were “yes,” no,” or “don’t know.”

^¶¶^ Flavor type use among current (past 30-day) users of flavored e-cigarettes was determined by answers to the question, “What flavors were the e-cigarettes that you have used in the past 30 days? (Select one or more).” Response options were “menthol,” “mint,” “clove or spice,” “fruit,” “chocolate,” “alcoholic drinks (such as wine, margarita, or other cocktails),” “candy, desserts, or other sweets,” and “some other flavor not listed here.” Those who selected “some other flavor not listed here” could provide a write-in response; write-in responses corresponding to an original response option were recoded.

Among current youth e-cigarette users overall, 84.7% used flavored e-cigarettes, including 85.8% of high school users and 79.2% of middle school users. Among all current flavored e-cigarette users, the most commonly used flavor types among both middle and high school students were fruit, followed by candy, desserts, or other sweets; mint; and menthol. When examined by device type used, the most commonly used flavor types among current flavored disposable e-cigarette users were fruit (78.7%; 760,000); candy, desserts, or other sweets (34.3%; 330,000); mint (30.1%; 290,000); and menthol (21.5%; 200,000). The most commonly used flavor types among current flavored pod or cartridge users were fruit (57.9%; 270,000); menthol (46.3%; 210,000); mint (30.7%; 140,000); and candy, desserts, or other sweets (28.2%; 130,000). The most commonly used flavor types among current flavored tanks or mod systems users were fruit (70.9%; 100,000); candy, desserts, or other sweets (51.2%; 70,000); mint (34.5%; 50,000); and menthol (24.7%; 30,000). Among current flavored e-cigarette users, fruit was the most commonly reported flavor type overall, by school level, and across all e-cigarette devices.

The 2021 NYTS was fully conducted amid the global COVID-19 pandemic, during which time eligible students could participate in the survey in classrooms, at home, or at some other place. Differences in tobacco use estimates by location^††^ might be due to potential underreporting of tobacco use behaviors or other unmeasured characteristics among youths participating outside of the classroom. Thus, estimates from the 2021 NYTS should not be compared with previous NYTS survey waves that were primarily conducted on school campuses. 

Approximately 2.06 million youths were estimated to be current e-cigarette users in 2021. Use of tobacco products by youths in any form, including e-cigarettes, is unsafe. Most e-cigarettes contain nicotine, and nicotine exposure during adolescence can harm the developing brain (*5*). Ongoing efforts to address youth e-cigarette use, including FDA’s prioritized enforcement against certain unauthorized flavored, cartridge-based e-cigarettes in 2020, are critical (*4*). As the tobacco product landscape continues to evolve, sustained implementation of comprehensive tobacco control and prevention strategies at the national, state, and local levels, coupled with FDA regulation, can reduce and prevent tobacco product initiation and use among youths (*5*).

## Footnotes

* There are a variety of different types of e-cigarette devices that are currently available. Disposable e-cigarettes come prefilled with e-liquid, and the entire device is designed to be discarded after a single use. Other devices have “pods” or “cartridges” that hold the e-liquid. Some pods or cartridges come pre-filled with e-liquid and are replaced after use, while others can be refilled by the user. Tank or mod-type devices can also be refilled by users, but are also usually customizable, allowing the user to change the temperature or voltage, nicotine concentrations, and add accessories to enhance the user experience.

† The final sample consisted of 508 schools, 279 (54.9%) of which participated; among 25,149 students, 20,413 (81.2%) students participated. The overall response rate (44.6%) is the product of the school-level and student-level participation rates. https://www.cdc.gov/tobacco/data_statistics/surveys/nyts/index.htm

§ Because of state and local COVID-19 protocols (e.g., distance or hybrid learning, restrictive travel, or visitor access), the 2021 NYTS data collection was transitioned from an in-person, tablet-based administration to a fully online administration. Eligible students could participate in classrooms, at home, or in some other remote learning environment. Overall, 50.8% of students who completed the 2021 NYTS reported completing the survey in a school building or classroom and 49.2% at home or at some other place. Because of these differences in data collection procedures, the 2021 NYTS estimates should not be compared with previous NYTS survey waves that were primarily conducted on school campuses.

¶ Weighted population estimates were rounded down to the nearest 10,000 students.

** 45 C.F.R. part 46; 21 C.F.R. part 56.

†† Youths who reported participating in the 2021 NYTS in a school building or classroom reported a higher prevalence of e-cigarette use compared with youths participating at home or at some other place; 15.0% of high school students who took the survey in a school building or classroom reported currently using e-cigarettes compared with 8.1% of those who took the survey at home or at some other place (p <0.001).
